# Toddler with giant omental cyst, profound anemia, and shock: case report and review of the literature

**DOI:** 10.3389/fmed.2023.1255545

**Published:** 2023-09-28

**Authors:** Fengchun Cheng, Xueling Xing, Xiaoming Liu, Shuai Sun, Zhaona Lv, Xiaoliang Xu, Tingliang Fu, Lei Geng

**Affiliations:** ^1^Department of Pediatric Surgery, Binzhou Medical University Hospital, Binzhou, Shandong, China; ^2^Department of Radiology, Binzhou Medical University Hospital, Binzhou, Shandong, China; ^3^Pediatric Intensive Care Unit, Binzhou Medical University Hospital, Binzhou, Shandong, China

**Keywords:** omental cyst, hemorrhage, complication, shock, pediatric, case report

## Abstract

Giant greater omental cysts with associated massive hemorrhage are rare. We encountered a 16-month-old boy with a four-day history of acute abdominal pain, distension, and paleness. Physical examination revealed a blood pressure of 74/27 mmHg. No well-defined masses were observed on abdominal palpation. The hemoglobin level on admission was 24 g/L. After initial resuscitation and blood transfusion, a computed tomography (CT) scan was performed, revealing a giant cystic mass with an intracystic hemorrhage. The diagnosis was confirmed via exploratory laparotomy, and the cyst, with the attached partial omentum was removed. Pathological findings revealed a simple cyst originating from the greater omentum. The patient recovered uneventfully and remained well during the two-year follow-up period. We reviewed the literature published over the last 27 years on cases of omental cysts to evaluate demographic characteristics, clinical presentations, complications, diagnostic tool options, and surgical approaches.

## 1. Introduction

Intra-abdominal cysts, including mesenteric cysts, omental cysts, retroperitoneal lymphangiomas, and intestinal duplication cysts, are rare in the pediatric population. The estimated incidence is 1 in 20,000 admissions to a pediatric hospital (1–3) and among these, omental cysts are rare, representing 10%–30% of cystic lesions (4). The clinical manifestations of omental cysts include a painless mass incidentally found, moderate or marked abdominal distension, acute or chronic abdominal pain, fever, vomiting, and anemia (1, 5–10). Giant omental cysts may mimic ascites for years and may persist for several months after treatment for misdiagnosed abdominal tuberculosis (1, 7, 8, 10). Several cases have presented with severe anemia and shock, requiring fluid resuscitation and massive blood transfusion (5, 6, 11, 12). Herein, we describe a new case of giant omental cyst complicated by spontaneous massive hemorrhage that presented with profound anemia and shock, which was successfully treated, with a favorable outcome. The literature related to omental cysts from 1996 to date was reviewed and analyzed. As shown in Table 1, more than half of the patients were between one and seven years old. The main presentations were abdominal distension mimicking ascites (36.8%), abdominal masses (25%), anemia (13.2%), and acute abdominal pain (13.2%). Omental cysts should therefore be a diagnostic consideration in all children presenting with abdominal pain. We aimed to increase awareness of this rare disorder, explore its characteristics, and suggest strategies for diagnosis and management.

**Table 1:** The clinical characteristics of patients with omental cysts in the literature (*n* = 68).

Variable	Number of cases (*n*)	Percent
Age at admission		
Neonates and infants (1d – ≤ 1y)	9	13.2
Pre-school (1y < age ≤ 7y)	39	57.3
School (7y < age ≤ 18y)	9	13.2
N/A	11	16.2
Gender		
Male	33	48.5
Female	24	35.3
N/A	11	16.2
Initial presentation and diagnosis		
Ascites	25 (including tuberculous ascites, *n* = 4; paracentesis, *n* = 5; anti-TB, *n* = 2)	36.8
Abdominal mass	17	25.0
Acute abdomen	9 (including peritonitis, *n* = 6; appendicitis, *n* = 2; SBO, *n* = 1)	13.2
Anemia	9 (including the present case)	13.2
Ovary cyst	4 of 25	16.0
N/A	8	11.8
Complications	27	39.7
Hemorrhage	18	26.5
Torsion	4	5.9
Infection	3	4.4
SBO	1	1.5
Bowel perforation	1	1.5
Imaging investigation		
USG	57	83.8
CT	37 (repeated CT, *n* = 1)	54.4
MRI	11	16.2
GI series	4	5.9
N/A	9	13.2
Cyst size (cm)		
≤ 10	13	19.1
11 < size ≤20	22	32.4
>20	13	19.1
N/A	20	29.4
Surgical approach		
Laparotomy	45	66.2
Laparoscopy	22 (converted to laparotomy, *n* = 3)	32.4
Minilaparotomy via umbilicus	1	1.5
Pathologic findings		
Lymphangioma	50	73.5
imple cyst	17	25.0
Inflamed melanotic cyst	1	1.5
Outcome and follow-up		
Cure without recurrence	68	100

TB, tuberculosis; SBO, small bowel obstruction; GI, gastrointestinal; USG, ultrasonography; CT, computed tomography; MRI, magnet resonance imaging; N/A, not available.

## 2. Case presentation

**Figure 1:**
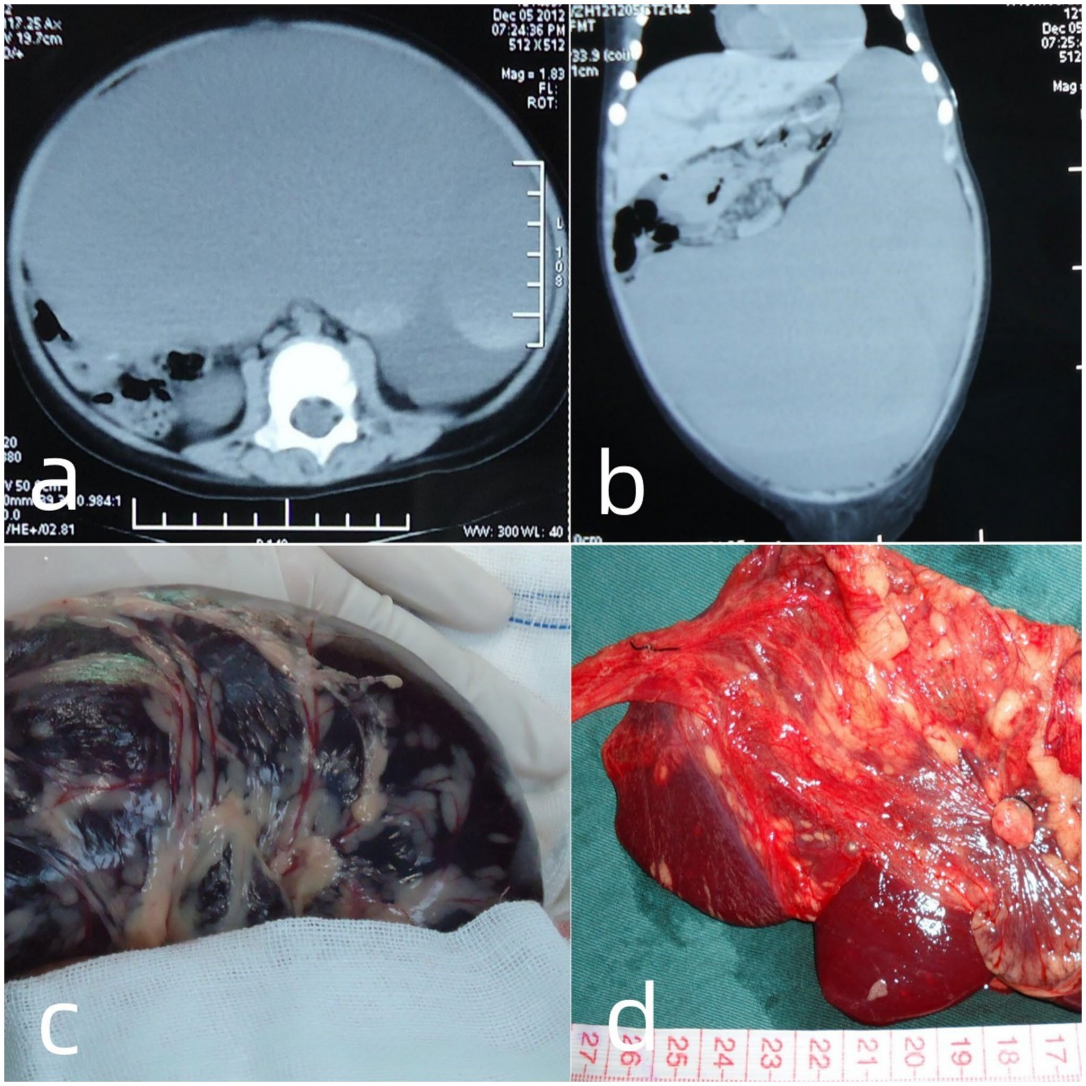
Abdominal and pelvic CT scans, revealing a large hypodense lesion that extended from the subdiaphragmatic space to the pelvis **(A,B)**. Intraoperative findings showing a well-defined cyst containing a large volume of fresh hemorrhagic fluid **(C)**. The surgically excised specimen showing a decompressed cyst, with attached partial greater omentum **(D)**.

A previously healthy 16-month-old boy presented with a four-day history of acute abdominal distension and profound anemia. His parents denied any preceding trauma or bleeding disorders. Upon physical examination, he was pale and lethargic, with tachycardia of 162 beats per minute. His blood pressure was 74/27 mmHg, with cool extremities, prolonged capillary refill, and flat neck veins. His oxygen saturation was 98%–100% on room air; the patient was tachypneic. He presented with severe abdominal distension; however, no well-defined abdominal mass was palpated. Initial laboratory data showed profound anemia, with hemoglobin 24 g/L, red blood cell count 1.03 × 10^12^/L, and hematocrit 7.8%. The white blood cell count was 9.5 × 10^9^/L and the platelet count was 304 × 10^9^/L. Other laboratory parameters, including coagulation screening and serum chemistry, were within normal limits. The patient was immediately fluid resuscitated and received a massive blood transfusion, including 600 mL of concentrated red blood cells and 100 mL of fresh frozen plasma. The patient responded well to therapy and his hemoglobin level reached 92 g/L before surgical intervention. Point-of-care ultrasonography revealed a large anechoic mass with dense debris echoes, suggestive of hemorrhage into the cyst. A chest X-ray before surgery revealed no mass or hydrothorax. Abdominal computed tomography (CT) revealed a cystic unilocular hypodense lesion from the subdiaphragmatic space to the pelvis, measuring 18 × 17 × 10 cm (Figures 1A,B). Due to a suspicion of an intra-abdominal cyst associated with spontaneous massive bleeding, emergency surgical exploration was advised. Written informed consent for the surgical procedure was obtained from the guardian of the patient. Intraoperative findings revealed a large thin-walled and well-defined cyst that originated from the greater omentum and contained fresh hemorrhagic fluid (Figure 1C). Intraperitoneal bleeding was not observed. After controlled decompression using an aspirator, the cyst, with the attached greater omentum, was completely excised (Figure 1D). Hemostasis was achieved using bipolar electrocoagulation ligation of the feeder vessels. No abdominal drainage was required. Pathological examination of the excised specimen revealed a unilocular cyst lined by mesothelial cells and the absence of smooth muscle, confirming the diagnosis of a simple mesothelial cyst of the omentum. The postoperative recovery was uneventful, and the patient was discharged on the ninth postoperative day. An ultrasound scan was normal at the two-year follow-up after surgery.

## 3. Discussion

Omental cysts are uncommon congenital cystic lesions that rarely occur in children (1–4). A giant omental cyst complicated by spontaneous massive hemorrhage is an extremely rare but life-threatening condition (5, 6, 13, 14). Previously reported cases of giant omental cystic lymphangioma were associated with massive hemorrhage (5, 13, 14). In the present case, the patient had a progressively distended abdomen, profound anemia, and unstable hemodynamics. We reviewed the literature published over the last 27 years on cases of omental cysts by evaluating demographic characteristics, clinical presentations and complications, diagnostic tool options, differential diagnosis, and surgical approaches. Our analysis was conducted using the keywords “omental cyst,” “mesenteric cyst,” and “abdominal cystic lesions, pediatric” in the PubMed^®^ database (Table 1) (1, 5, 6, 8–11, 13–40).

Based on the literature review, the results of the pathological diagnosis (41) revealed lymphangioma (*n* = 50, 73.2%), simple cysts (*n* = 17, 25%), and inflammatory melanotic cysts (*n* = 1, 2.8%). In the present case, the pathological findings revealed a simple unilocular cyst lined with mesothelial cells.

Clinically, an abdominal mass may mimic an ovarian cyst in female children (16%). Of the 68 reported cases, 27 (39.7%) patients experienced complications, including massive hemorrhage (26.5%), torsion (5.9%), infection, small bowel obstruction, and intestinal perforation. Profound acute anemia is usually associated with massive intracystic hemorrhage (5, 6).

In association with the absence of specific symptoms, the rarity of omental cysts, especially giant cysts, may complicate diagnosis. Greater omental cysts may mimic ascites (1, 7, 19, 22, 40), ovarian cysts (26, 27, 29, 42), acute abdomen (7, 25, 26, 42), and anemia (5, 6). Omental cysts should be considered as a diagnostic hypothesis in children with abdominal pain, with or without a mass (42). In the absence of trauma or hematologic disorders, a diagnosis of profound pediatric hemorrhagic anemia with severe abdominal distension and shock should rule out a giant omental cyst complicated by spontaneous major bleeding (14).

An accurate preoperative diagnosis may result in appropriate management options (24). Imaging is of paramount importance in the differential diagnosis of intra-abdominal cystic lesions (35). Regarding the imaging tool option, an initial ultrasonography scan, which reveals the cystic characteristics of the lesion in four-fifths of cases, was used as a diagnostic tool (18, 27, 43). CT and MRI scans were chosen in 54.4% and 16.2% of cases, respectively, which provided further and more accurate information, including the location, size, content, and relationship of the cyst with adjacent structures (8, 16, 21, 30). Although imaging studies, including point-of-care ultrasonography, CT scan, and magnetic resonance imaging, are vital for the diagnosis of intra-abdominal cysts, they may fail to differentiate giant omental cysts from other cystic lesions or ascites, such as mesenteric cysts (7), intestinal duplication cysts (17, 37), and abdominal tubercular ascites (8). It is necessary to do a chest CT and to detect tumor biomarkers to rule out other rare conditions, especially in patients with stable hemodynamic status (44, 45); however, increasing concern regarding radiation exposure and other potential risks from CT scan in babies should be considered (46, 47).

Regarding the management of patients with massive intracystic hemorrhage, treatment should focus on rapid crystalloid resuscitation followed by blood transfusion to restore blood volume. Emergent surgery is indicated due to high intra-abdominal pressure and the risk of continued bleeding or further major bleeding in a short timeframe, especially in toddlers (5, 6, 14).

Most authors agree with complete excision of the cyst without omentectomy or partial omentectomy to prevent the potential risk of complications due to the cyst (48). In selected cases, several approaches, including traditional open surgery (*n* = 45, 66.2%) (11, 22, 32), laparoscopic surgery (*n* = 22, 32.4%) (16, 18, 32, 39), and transumbilical minilaparotomy (30), have been described for the treatment of omental cysts. When technically available, laparoscopic surgery is usually preferred because of its minimal invasiveness, reduced blood loss and postoperative ileus, less postoperative pain, shorter hospitalization, and faster return to normal activity (8, 9, 15, 49).

It has been reported that the creation of a low-pressure pneumoperitoneum without compromising the cardiovascular physiology is possible for large intra-abdominal cystic lesions (50). Controlled decompression by aspiration of a large cyst can provide adequate space for manipulation (50–52). It is important to excise cystic lesions completely laparoscopically, especially large omental cysts. However, careful patient selection for the choice of laparoscopic approach is necessary and conversion to laparotomy is possible if required from a technical standpoint (49, 53).

In conclusion, omental cysts are rare. The diagnosis may be difficult because of nonspecific symptoms. Complete open or laparoscopic cyst removal is recommended to prevent potential complications and recurrence. We present the case of a toddler with a giant omental cyst complicated by a spontaneous massive hemorrhage leading to profound anemia and shock. Our case study reveals the importance of early recognition and proper management of this potentially fatal complication in patients with giant omental cysts.

## Data availability statement

The raw data supporting the conclusions of this article will be made available by the authors, without undue reservation.

## Ethics statement

Ethical approval was not required for the study involving human samples in accordance with the local legislation and institutional requirements because (reason ethics approval was not required). Written informed consent for participation in this study was provided by the participants’ legal guardians/next of kin. Written informed consent was obtained from the individual(s) for the publication of any potentially identifiable images or data included in this article. Written informed consent was obtained from the participant/patient(s) for the publication of this case report.

## Author contributions

FC: Data curation, Methodology, Software, Validation, Writing – original draft, Writing – review & editing. XuX: Data curation, Supervision, Writing – original draft, Writing – review & editing. XL: Data curation, Methodology, Writing – original draft, Writing – review & editing. SS: Data curation, Methodology, Writing – original draft, Writing – review & editing. ZL: Data curation, Methodology, Writing – review & editing. XiX: Data curation, Methodology, Writing – original draft, Writing – review & editing. TF: Conceptualization, Supervision, Writing – original draft, Writing – review & editing. LG: Data curation, Investigation, Methodology, Resources, Supervision, Validation, Writing – review & editing.
